# Parkinson’s disease, dopaminergic drugs and the plant world

**DOI:** 10.3389/fphar.2022.970714

**Published:** 2022-09-05

**Authors:** Peter Kempster, Andrew Ma

**Affiliations:** ^1^ Neurosciences Department, Monash Medical Centre, Clayton, VIC, Australia; ^2^ School of Clinical Sciences of Medicine, Monash University, Clayton, VIC, Australia

**Keywords:** Parkinson’s disease, plants, dopamine, levodopa, phytochemicals

## Abstract

A large proportion of drugs used for the treatment of neurological disorders relate to naturally occurring compounds, many of which are plant alkaloids. This is particularly true of Parkinson’s disease (PD). The pharmacopoeia of PD has strong botanical origins, while major discoveries about the neurochemistry of the basal ganglia came from the study of phytochemicals. This article narrates the development of pharmacotherapy for PD in terms of historically important plant-derived substances—tropane and hamala alkaloids, reserpine, levodopa, apomorphine, and ergoline dopamine receptor agonists. Alkaloids are nitrogen-containing secondary metabolic products that tend to be biologically active. They appear to be involved in plants’ adaptation to herbivorous animals, though their exact purpose and the ways in which they work are uncertain. A sizable group of alkaloids influence animal dopaminergic systems, highlighting a key biological relationship. While animals must acquire the energy that plants harness, plants need to engage with the animal attribute that they lack—movement—in order to maximize their reproductive fitness. Neuroactive flowering plant compounds have been interacting with vertebrate and invertebrate motor systems for 100 million years. A deep evolutionary connection helps to explain why the pharmacological treatment of PD is imprinted with the power of these mysterious botanical chemicals.

## Introduction

Well before the modern era of neuropharmacology, physicians had a special sense that drugs prepared from plants might alleviate the disability of Parkinson’s disease (PD). Jean-Martin Charcot (1825-93) wasn’t the first to try such treatments but, having refined James Parkinson’s description of the disorder, his use of the alkaloid hyoscyamine may have been the first documented anti-parkinsonian drug effect (Ordenstein, 1867). Botanical substances later signposted the way to understanding the neurochemistry of the basal ganglia, culminating in the miraculous advent of levodopa.

The motor deficits of PD are caused by progressive loss of dopaminergic neurons in the substantia nigra pars compacta. These neurons project to dopamine D1 and D2 receptors in the striatum. Symptomatic treatments address the striatal dopamine deficiency in several ways—direct stimulation of dopamine receptors (levodopa, dopamine receptor agonists), altering the balance between dopamine and acetylcholine in the striatum (muscarinic acetylcholine receptor antagonists), blocking enzymes that degrade dopamine (monoamine oxidase, catechol-O-methyl transferase inhibitors). Plant secondary products in each of the categories have been used to treat PD. The most effective of all treatments of the disorder, levodopa, is a plant chemical with the ability to reach the brain and to replenish dopamine. These things have probably not happened by chance, and it may be in the interests of plants to be able to influence the dopaminergic systems of animals. This narrative about pharmacological therapy for PD will highlight less familiar relationships between the human motor system and the plant kingdom in two ways—by examining the roles of plants in the history of antiparkinsonian drug treatment; and by reviewing aspects of the biology of plant secondary metabolic products.

## Plant alkaloids

Alkaloids, which are the major natural medicinal source, are an incompletely understood aspect of plant chemistry. They are cyclic organic compounds in which at least one non-carbon atom is nitrogen (Pelletier, 1983). Most are biologically active. Alkaloids can be classified from the nomenclature of their ring chemistry, or according to the amino acid from which they are derived (Aniszewski, 2007). Many contain aromatic rings assembled from proteinogenic amino acids phenylalanine, tyrosine or tryptophan. Only plants, fungi and microorganisms have the capacity to create aromatic compounds, through a 7-step enzymic sequence, the shikimate pathway (Tohge et al., 2013). In plants, this is a high-volume process that requires considerable energy. Lysine, another essential amino acid for animals with its own synthetic machinery in plants, and ornithine are the main sources of alkaloids with non-aromatic rings (Galili et al., 2016). The complex enzyme systems associated with these pathways have the capacity to generate a wide variety of carbon molecular skeletons, and more than 50,000 secondary metabolic products have been identified, of which alkaloids are the most important group (Wink, 2020). Plant species differ in the richness of their alkaloid content, but most contain a mixture of these chemicals, diverse in both their molecular structure and the biology of their effects. Alkaloid levels in individual plants vary with part, life cycle and season (Waller and Nowacki, 1978). Climate and other environmental factors affect alkaloid profile across populations of the same species (Selmar and Kleinwächter, 2013). Other small and medium-sized molecules that do not contain nitrogen, such as terpenoids and polyphenols, may play similar roles (Wink, 2020).

Although their complexity and wide prevalence amongst plants is well established, the purpose of alkaloids remains elusive. Explanations that they are just superfluous metabolic by-products, or auxiliary storage reservoirs for nitrogen, seem unsatisfactory. Some resemble plant growth hormones in their molecular structure, though effects on growth have not convincingly been shown (Waller and Nowacki, 1978). While certain alkaloids have activity against fungi, bacteria or viruses, it is not apparent that alkaloid-rich plants enjoy significant advantages over alkaloid-poor ones when it comes to infective agents (Waller and Nowacki, 1978). The most widely advanced theory is that alkaloids are part of plants’ defences against herbivorous animals (Wink, 2003).

The pharmacological treatment of neurological disorders depends quite strongly on plant alkaloids. A recent review article estimates that 84% of drugs approved for central nervous system indications relate to naturally occurring chemicals (Bharate et al., 2018). Nearly all can be allocated to one or other of 20 classes based on natural molecular scaffolds. Eighteen scaffolds are of plant origin, and most come from alkaloids. There may be several reasons for this aspect of neuropharmacology. Historically, the pharmaceutical industry grew out of traditional knowledge about plant medicines. Molecular features common to the natural scaffolds may increase affinity with receptor binding sites and other target proteins. Compared with purely synthetic agents, the natural structures confer greater chirality (giving rise to distinct D- and L- stereoisomers) and more rigidity (because of ring conformations) (Bharate et al., 2018). These molecules are also better able to cross the blood-brain barrier. Natural product and natural product-inspired drugs may have their privileged influence on neurotransmitter systems because of evolutionary relationships between plants and animals.

## Prehistory of dopaminergic treatment

### Anticholinergic alkaloids

Large, pharmacologically potent amounts of anticholinergic tropane alkaloids (atropine, hyoscyamine, hyoscine) are present in some Solanaceae (potato) family species: *Atropa belladonna* (deadly nightshade), *Hyoscyamus niger* (henbane), *Datura stramonium* (thorn apple or jimsonweed). Early human hunter-gatherers must have identified the toxicity of these plants, and they were well known to medical authorities of the ancient world for narcotic, analgesic and hallucinatory properties. Henbane was added to strengthen beer in the Middle Ages.

**FIGURE 1:**
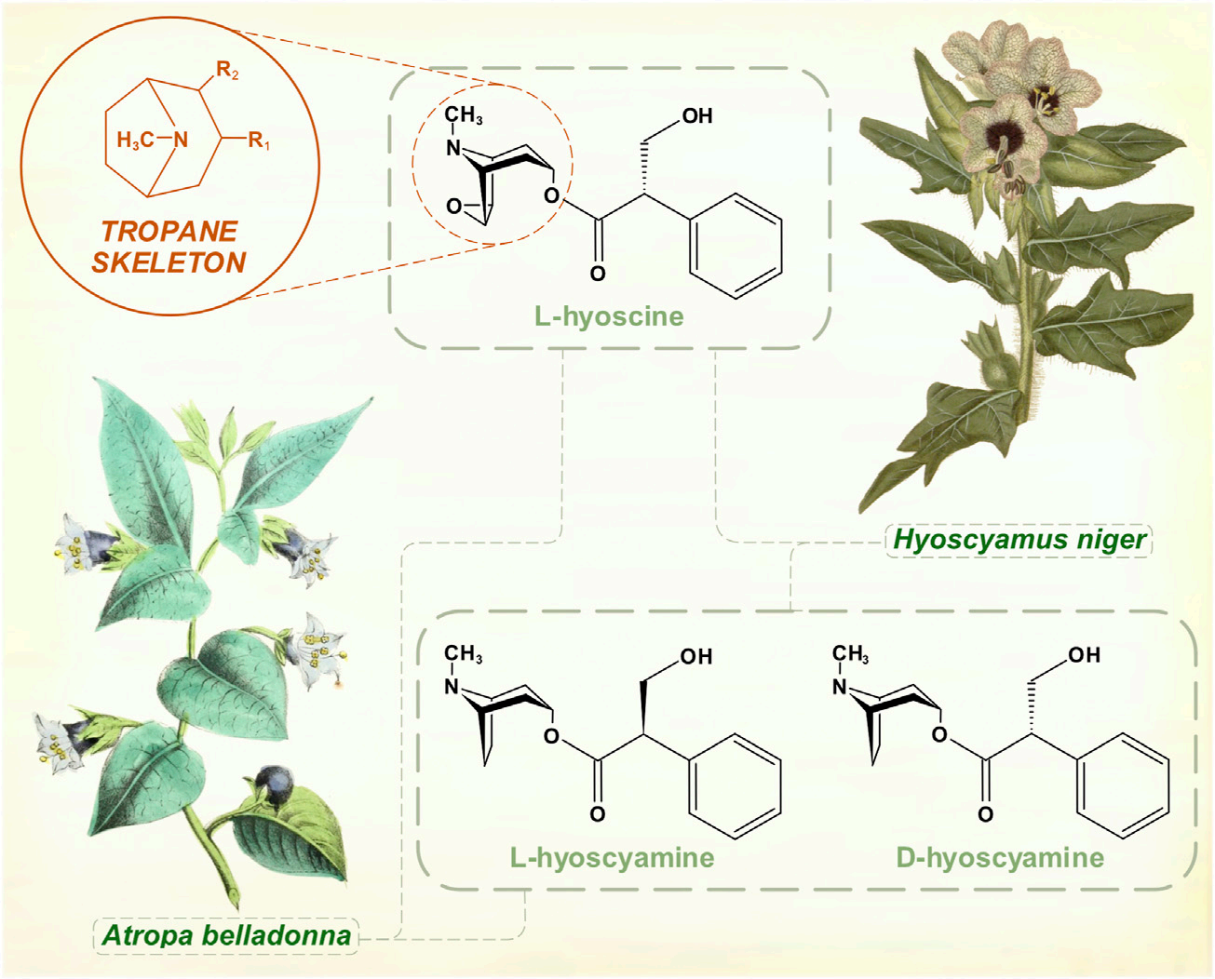
The muscarinic anticholinergic tropane alkaloids. Atropine is the racemic mixture of L- and D-hyoscyamine. [Illustrations adapted from Strong (Strong, 1855a) and Curtis et al. (1822), both available in the public domain].

Atropine had been purified in 1833 and its empirical formula was worked out (Geiger, 1833). In Charcot’s time, these alkaloid preparations were associated with the source of their original isolation—atropine from *Atropa belladonna*; hyoscyamine from *Hyoscyamus niger*. What he called hyoscyamine probably contained a mixture of the active tropane alkaloids hyoscyamine, atropine and hyoscine, though hyoscyamine is the dominant chemical in many of these species. Hyoscine was isolated from henbane in 1880 (Ladenburg, 1880a). Originally thought to be an isomer of atropine, its distinct chemical formula was determined at around the same time that the isomeric relationship between atropine and hyoscyamine (racemic mixture and levo-rotatory form of the same molecule) was worked out (Ladenburg, 1880b) (Figure 1). The alkaloid scopolamine was extracted from *Scopolia* (another nightshade genus) plants in 1888 (Schmidt and Henschkem, 1888). Several years elapsed before it was widely recognized as identical to hyoscine, long enough for a synonymous nomenclature to develop. Once reliable preparations of the salts of hyoscine were developed by the pharmaceutical company Merck, Wilhelm Erb advocated its use in PD (Erb, 1887). Atropine extracted from *Atropa belladonna* and hyoscine from *Duboisia* (corkwood) species eventually supplanted hyoscyamine for most therapeutic purposes. Hyoscyamine is less stable, tending to racemize to atropine during and after extraction. While preparations of these tropane alkaloids were generally held to have efficacy, the benefits were modest and other treatments were by no means overshadowed by them. Charcot documented the variety of other therapies for PD in use during the 1870s—salts of arsenic, barium, bromine, iron and silver; botanical alkaloids such as strychnine, ergot and physostigmine; galvanic treatments (Charcot, 1877). Gowers’ Manual of Diseases of the Nervous System mentions ‘hyoscyamin’ in a generally pessimistic inventory of existing treatments (Gowers, 1886), which also included morphia, conium (*Conium maculatum*, or hemlock) and Indian hemp.

Atropine became the dominant anticholinergic during the first half of the 20th century, administered as belladonna extract, or as purified atropine sulphate of standard potency. It had the reputation among the anticholinergic alkaloids of the widest margin of safety (Goodman and Gilman, 1955). Higher dosage regimes with gradual titration were developed (Römer, 1932). A “Bulgarian treatment” based on belladonna root preparations of high but variable alkaloid content was promoted during the 1930s (Neuwahl and Fenwick, 1937). These were the years following the encephalitis lethargica pandemics, and atropine appeared to have greater efficacy, and to be better tolerated, in post-encephalitic parkinsonism than in idiopathic PD (Römer, 1931, 1932). These treatments clearly gave more benefit than had been witnessed by Charcot and Gowers. Still, Kinnier Wilson’s textbook, published posthumously in 1940, listed the various Solanaceae alkaloid preparations—belladonna, stramonium, hyoscyamus, scopolamine, atropine—without a great deal of enthusiasm (Wilson, 1940). He referred to a combination cited by Charcot, ergot and belladonna, which might have been in wider usage for perceived synergistic effects. Benzhexol, which emerged in the late 1940s from research into the pharmacological properties of piperidine compounds, was the first synthetic anti-parkinsonian agent to be widely prescribed (Doshay et al., 1954).

### The hamala alkaloids

**FIGURE 2:**
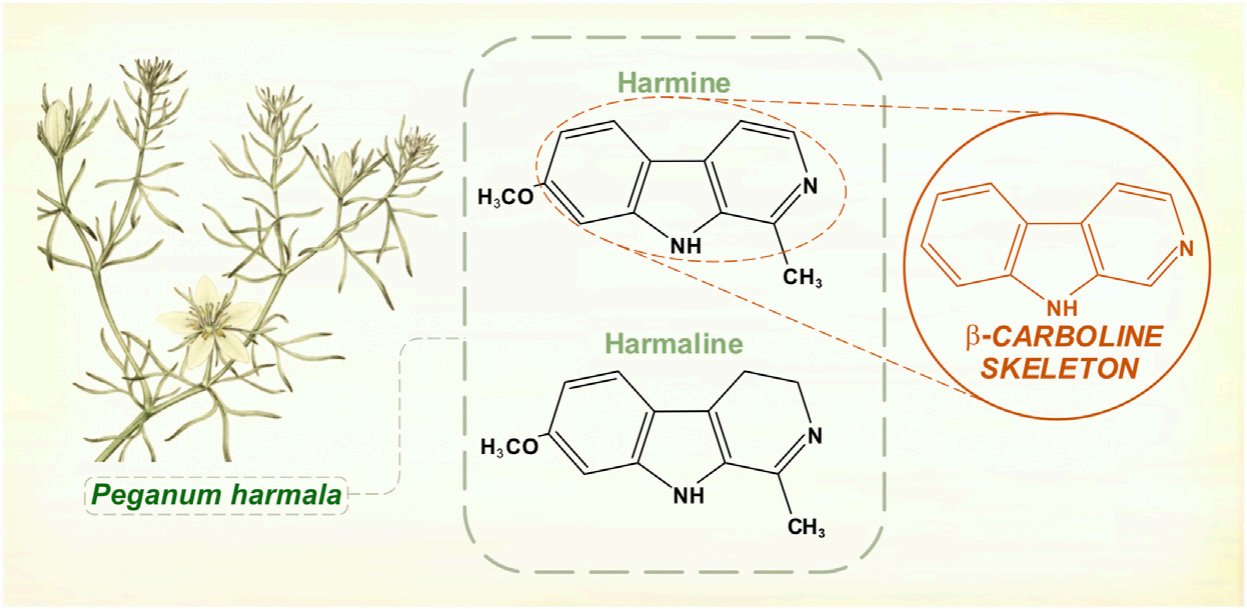
Hamala alkaloids contain a β-carboline structural skeleton. Harmine and harmaline are reversible inhibitors of monoamine oxidase (monoamine oxidase-A is selectively affected). [Illustration adapted from Bulliard (1780), available in the public domain].

Ancient Greek physicians wrote about medicinal effects of *Peganum harmala*, the Asian or Syrian rue plant. In the first half of the 19th century, two chemicals were isolated from its seed pods—harmine and harmaline (Goebel, 1841; Fritzsche, 1847) (Figure 2). Test doses given to animals caused convulsions and autonomic instability (Flury, 1911).

Explorers of the Amazonian rain forests had documented the potent hallucinatory effects of yagé, a drink prepared by indigenous peoples from the vine *Banisteriopsis caapi* and used in ritualistic and shamanic practices (McKenna et al., 1998; Brabec de Mori, 2011). The Columbian chemist Barriga Villalba published in 1925 on an alkaloid extracted from *Banisteriopsis caapi*, which he called “yajéine” (Barriga Villalba, 1925). The substance found its way to European pharmaceutical laboratories, where it acquired the alternative name banisterine. It was soon realized that banisterine and harmine shared an empirical formula and physical properties, although it took several years before it was generally accepted that they were chemically identical (Wolfes and Ivers, 1929). Echoing the hyoscine/scopolamine controversy of 40 years before, there was skepticism that such different, geographically separate plants would be producing the same alkaloid. Furthermore, extracts from *Peganum harmala* had never been shown to possess the florid hallucinogenic properties of yagé. In fact, the South American brew also contained leaves of other species rich in *N,N*-dimethyltryptamine (McKenna et al., 1998). It was later shown that harmine and harmaline were reversible monoamine oxidase inhibitors (Udenfriend et al., 1958). By this action, *Banisteriopsis caapi* permitted dimethyl tryptamine’s psychoactive serotonergic effects.

Merck had been interested in commercial possibilities of these alkaloids for some time, and its laboratories conducted basic chemical and toxicological research. In the late 1920s, the company distributed quantities of harmine for single dose clinical experiments. Chance observations about motor effects in high-dose animal studies led to trials in parkinsonian subjects, and some striking benefits were observed (Beringer, 1928). Rigidity and hypokinesia improved more than tremor, and post-encephalitic parkinsonism appeared to respond better than PD (Lewin, 1928; Frank and Schlesinger, 1930). The chemical equivalence of banisterine and harmine meant that expeditions to the Amazon were not needed, and that large quantities could be processed from *Peganum harmala*, a hardy and invasive species easily sourced right around the Mediterranean basin. Further trials of oral harmine confirmed anti-parkinsonian effects, but also highlighted limitations. Not all patients responded, and early treatment benefits were prone to wane (Rustige, 1929). Within a decade, harmine had fallen out of fashion. Most authorities were convinced of its inferiority to hyoscine and atropine (Halpern, 1931).

More recently, it has been shown that extracts of *Peganum harmala* also possess catechol-O-methyl transferase inhibiting properties, which reside with its harmaline alkaloid content (Yalcin and Bayraktar, 2010).

### Reserpine

**FIGURE 3:**
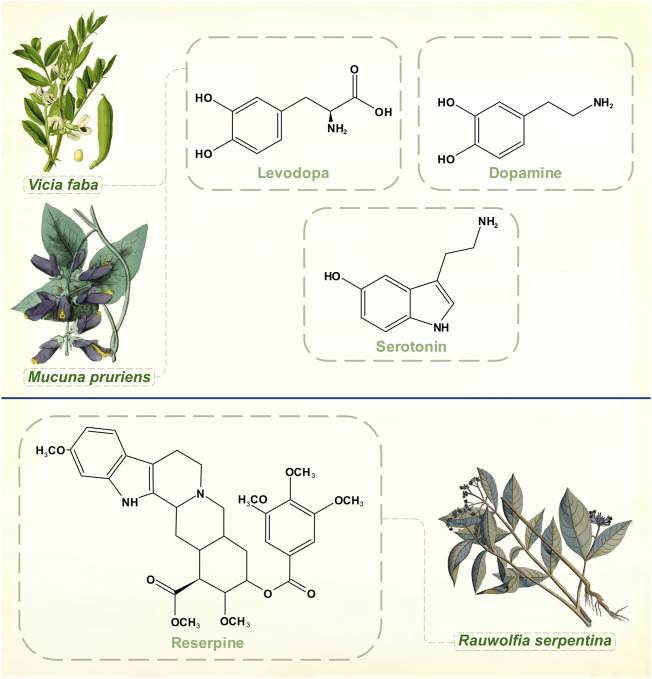
Dopamine, levodopa and serotonin. Experiments with reserpine, an irreversible inhibitor of vesicular monoamine transporters 1 and 2, played a key role in discoveries about monoamine neurochemistry. [Illustrations adapted from Van Rheede tot Drakenstein (1686), Thomé (1885) and Strong (1855b), all available in the public domain].

*Rauwolfia serpentina*, or Indian snakeroot, grows in the Indian subcontinent and South East Asia. Indian physicians were aware of its antihypertensive and sedative properties in the 1930s, and some calmative effects in psychiatric patients were noted around this time (Jain and Murthy, 2009). Reserpine, the alkaloid responsible for *Rauwolfia’s* ability to tranquillize, was identified by the Swiss pharmaceutical company Ciba (later merged into Novartis) in 1952 (Müller et al., 1952) (Figure 3). It was not until several decades later that reserpine’s mechanism of action as an irreversible inhibitor of vesicular monoamine transporter membrane proteins was fully elucidated (Near and Mahler, 1983; Scherman and Henry, 1984). The drug thus depletes noradrenaline, adrenaline, dopamine, serotonin, and histamine. Also in 1952, chlorpromazine was synthesized as a spin-off from anti-histamine research by Rhône-Poulenc (López-Muñoz et al., 2005). Soon, both agents were being used in psychiatric practice as effective antipsychotics. Motor symptoms with high doses of *Rauwolfia* extracts had been observed in India as early as 1944 (De, 1944/1945) and within a few of years of widespread psychiatric prescribing of reserpine it was reported that between 5 and 63% of patients developed parkinsonism (Kline and Stanley, 1955; Richman and Tyhurst 1955).

By 1960, reserpine was losing ground to chlorpromazine as a treatment of schizophrenia. Its clinical response was perceived to be slower in onset with more tendency to cause depression (Shepherd and Watt, 1956; Harris, 1957). Other synthetic antipsychotics such as the butyrophenones and benzamides were coming on to the market. Already, though, reserpine had made its indelible mark on neuropharmacology.

Links between serotonin and mental illness had already been suggested when Bernard Brodie, aware of reserpine’s clinical successes, remarked on its structural similarities with serotonin and lysergic acid diethylamide (all three molecules contain an indole heterocyclic group) (Shore et al., 1955). In 1955, he demonstrated that reserpine depleted serotonin in brain tissue, a crucial step in proving that serotonin was a neurotransmitter (Brodie et al., 1955).

Arvid Carlsson was then visiting Brodie’s laboratory at the National Heart Institute in Bethesda, Maryland, United States. He was interested in exploring the relationships between catecholamines and reserpine, though Brodie’s main focus remained with serotonin for the next few years. Despite accumulating clinical evidence of drug-induced extrapyramidal effects, scientists did not immediately appreciate that they had a chemical model of PD with reserpine, which was thought to produce general sedation of experimental animals rather than a specifically motor phenomenon. Carlsson’s body of Nobel Prize-winning research began with his 1957 observation that levodopa reversed the tranquillizing effect of reserpine in mice while 5-hydroxytryptophan didn’t (he had already understood that to increase catecholamine and serotonin levels in the brain it was necessary to administer their amino acid precursors) (Carlsson et al., 1957). He then showed that reserpine, when given to rabbits, caused almost complete disappearance of both dopamine and noradrenaline from the brain. The discovery that dopamine was differentially distributed from noradrenaline in both human and animal brains, with dopamine chiefly concentrated in the corpus striatum, pointed to an independent neurotransmitter function for dopamine (Carlsson, 1959). In Carlsson’s 1959 article it is clear that he had grasped both the motoric character of reserpine-induced deficits in animals and the hypokinesia of psychiatric patients treated with the drug. The conclusions that dopamine is involved in motor control, that large amounts of dopamine in the corpus striatum indicate an extrapyramidal function, and that its depletion by reserpine coincides with a syndrome resembling parkinsonism brought Carlsson to the threshold of discovering the neurochemical lesion of idiopathic PD. Ehringer and Hornykiewicz (1960) then found severe reductions of dopamine in parkinsonian brains, and Barbeau et al. (1961) reported that urinary dopamine excretion was depressed in patients with PD. Carlsson had already shown the way to circumvent the brain’s impermeability to dopamine.

## The miracle of levodopa

Carbocyclic amines and amino acids are not usually classified as alkaloids, though they broadly fit the definition. Levodopa is an example of a non-proteinogenic amino acid with a secondary metabolic product role in certain legume species. Markus Guggenheim, head of the pharmacological laboratory at Hoffman-La Roche, first isolated levodopa in 1913 (Guggenheim, 1913). He extracted it from seedlings of *Vicia faba* (broad beans), which he had picked from Fritz Hoffman-La Roche’s garden. He found that a dose of 2.5 g of the drug made him quite nauseated, but he could observe no biological activity in various experiments on rabbits. Guggenheim, who was blinded in a factory explosion in 1916, remained a faithful servant of Hoffman-La Roche until his retirement, but his conclusion that levodopa was inert saw the drug sidelined for decades (Guggenheim, 1961; Löffler, 1971).

### Early use of levodopa

The first clinical application of Carlsson’s discoveries was for extrapyramidal side effects of reserpine and phenothiazines. Degkwitz et al. (1960) found that levodopa produced moderate improvement in reserpine-induced parkinsonism and McGeer et al. (1961) thought that levodopa was less effective than the anti-histamine and mildly anti-cholinergic diphenhydramine for dystonia and parkinsonism caused by antipsychotics.

When levodopa was administered to patients with PD in single dose studies by intravenous (Birkmayer and Hornykiewicz, 1961) and oral (Barbeau et al., 1962) routes, it appeared to produce unequivocal improvement in motor function. More physicians experimented with the drug, and not all were as impressed by its therapeutic effects. In the mid-1960s, two double blind, placebo-controlled trials of levodopa were published. Fehling (1966) gave intravenous D,L-dopa to 27 parkinsonian patients, while McGeer and Zeldowicz (1964) gave both oral and intravenous levodopa doses to 10 individuals. Neither group of researchers could detect much difference between active and placebo treatment. In retrospect, these trials had probably been using doses of levodopa that were too low for periods that were too short.

### George Cotzias and practical oral levodopa therapy

George Cotzias, whose father had been mayor of Athens, came to the United States during World War II where he completed his medical training (Dole, 1996). Cotzias’s first PD study was based on a hypothesis about restoring neuromelanin from catecholamines (Cotzias et al., 1967). He administered D,L-dopa, melanocyte stimulating hormone and phenylalanine, and his discovery of the full potential of dopamine replacement therapy was really a by-product of the experimental objective. For his 1969 trial, Cotzias obtained levodopa, more potent than the racemic mixture that had often been used before (Cotzias et al., 1969). The main reason, though, for the success of this research was Cotzias’s belief that his patients would develop tolerance to the initial side effects of nausea and vomiting if the levodopa dose titration was gradual enough, and his patience and persistence to carry this policy through.

Cotzias’s 1969 paper showed the almost magical effects of starting levodopa treatment in patients who were disabled by advanced PD. He also observed that problems later associated with chronic levodopa therapy (Fahn, 1974; Marsden and Parkes, 1977)—motor fluctuations and dyskinesia—began almost immediately when patients with a severe dopaminergic deficiency were given the drug. More than 50 years later, levodopa remains the strongest, the most natural and, all things considered, the best therapy for PD.

### Naturally occurring levodopa in the treatment of PD

Two leguminous species contain substantial amounts of levodopa. Natural food sources present levodopa in a different physico-chemical form to tablets, which may have advantages for drug absorption and metabolism. *Vicia faba* has a culinary tradition in the Eastern Mediterranean area that probably dates back to Neolithic times. As Guggenheim reported, the pods are a richer source of levodopa than the beans themselves (Guggenheim, 1913). The fresh young beans, cooked in their pods with olive oil, have been reported to give more stable, satisfactory responses than tablets (Apaydin et al., 2000). In single dose studies with carbidopa co-administration, 25–50 g of bean mixture could outperform standard levodopa/carbidopa tablets on both clinical and pharmacokinetic grounds in an open-label study (Kempster et al., 1993).

The levodopa yield per weight of the tropical legume *Mucuna pruriens*, sometimes called the velvet bean, is considerably greater than from *Vicia faba* (Melvin et al., 1972). Administered without a decarboxylase inhibitor in a double blind comparison, *Mucuna* seed powder was more rapidly absorbed, produced higher levodopa levels, and caused no more side-effects than a standard levodopa/carbidopa preparation (Katzenschlager et al., 2004). Widely grown as a forage crop or to fix nitrogen in arable soils, *Mucuna pruriens* could be a practical alternative to commercially produced levodopa tablets in some circumstances (Fothergill-Misbah et al., 2020).

## Apomorphine

Arppe in 1845 was first to isolate this product of morphine (from *Papaver somniferum*) boiled with strong inorganic acid. The standard manufacturing process with hydrochloric acid was developed in 1869 by Matthiesen and Wright (Taba et al., 2013). They called it apomorphine to emphasize its differences from the opiate parent compound (Greek prefix apo-, away or apart from). Over the next few years, various motor effects were observed in animals, mainly induction of hyperkinetic repetitive behaviors (Harnack, 1874). It was used in medical and veterinary practice for its strong emetic powers. While aversion through emesis seems to have been a rationale for apomorphine treatment of alcohol and opiate addiction, some practitioners thought that its benefits did not rely purely on this principle (Dent, 1934).

**FIGURE 4:**
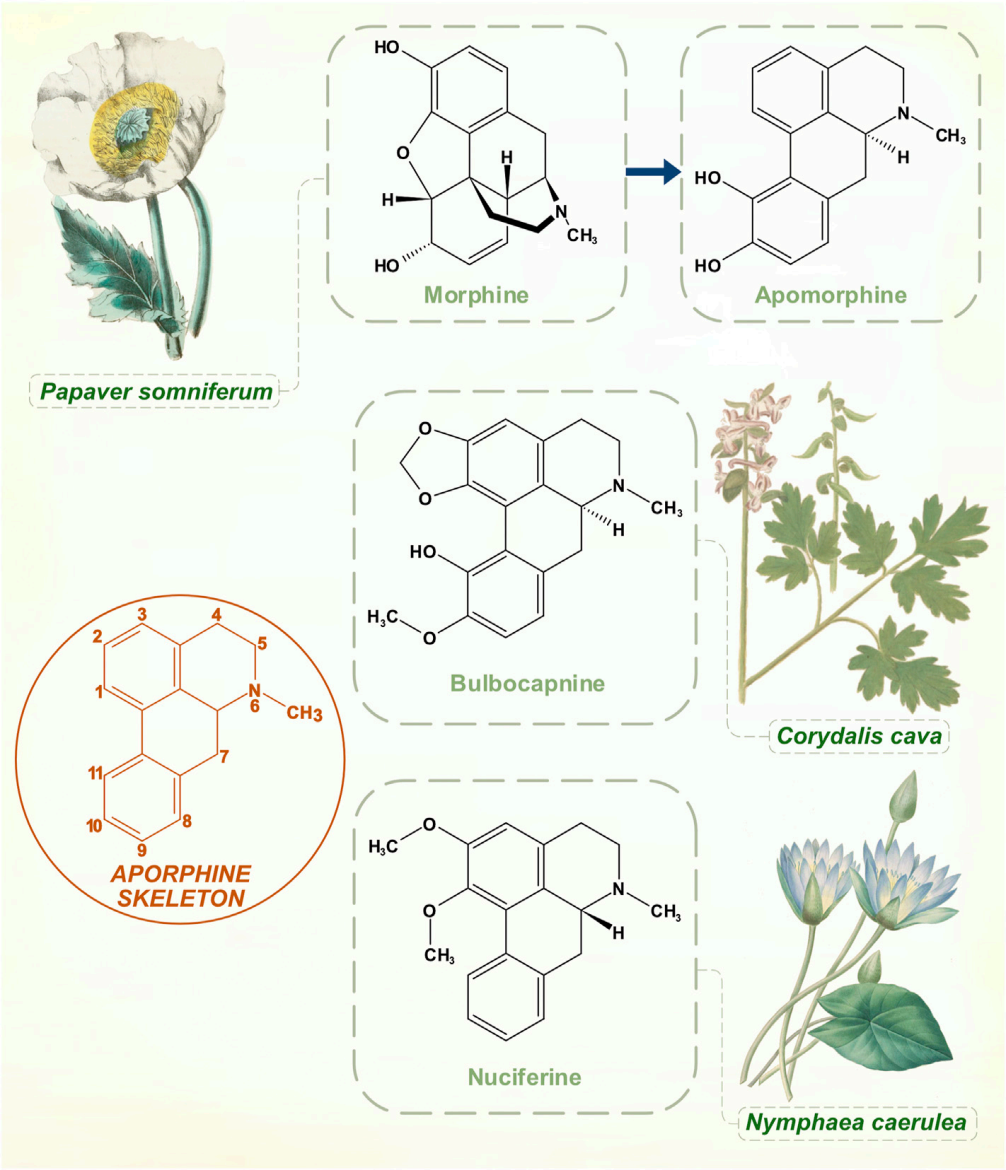
While boiling with concentrated hydrochloric acid is an ostensibly simple process, the transformation of morphine to apomorphine involves a complex sequence of reactions. Aporphine molecular skeleton shown with numbering. Naturally occurring aporphine alkaloids: bulbocapnine (*Corydalis* species) and nuciferine (*Nymphaea* species). Unlike apomorphine, both molecules are oxygenated at C1 and C2 positions. [Illustrations adapted from Strong (1855c), de Boodt (1596-1610) and Redouté (1833), all available in the public domain].

Narratives about the history of apomorphine in PD refer to its natural occurrence in *Nymphaea* (water lily) species, and on possible medicinal or ritualistic use of the drug by ancient civilizations (Taba et al., 2013; Djamshidian and Poewe, 2016). *Nymphaea caerulea*, the blue lotus plant of Northern Africa, appears as a motif in ancient Egyptian funerary art. Stone carvings and ceramics from the pre-Columbian Mayan culture show *Nymphaea ampla*, native to Central American freshwater lakes (Embolden, 1982; Bertol et al., 2004). Apomorphine belongs to the aporphine chemical family, which shares a benzoisoquinoline structural core. *Nymphaea* plants contain a number of these aporphine alkaloids. *Nymphaea* extracts are used for natural health and recreational purposes, marketed for benefits that include anti-anxiety, sleep-promoting, mildly psychoactive and aphrodisiac effects (Poklis et al., 2017). Apomorphine was identified in an analysis two of four proprietary e-cigarette substances purportedly prepared from *Nymphaea caerulea* (Poklis et al., 2017). Yet its presence in the plant is doubtful on chemical grounds, and apomorphine might be better classified as a derivative of a natural product. All other aporphine alkaloids found in plants are oxygenated at molecular positions 1 and 2, whereas neither of these carbons is substituted in apomorphine (Figure 4) (Neumeyer, 1985). No plant aporphine alkaloid has apomorphine’s potent dopamine receptor agonism. Bulbocapnine (found in *Corydalis* species), for instance, has structural resemblances to apomorphine but is only a partial agonist. Tried in the 1920s as a treatment for PD, bulbocapnine soon fell out of favor because of lack of efficacy and central side-effects (Fleischhacker, 1926; Leiner and Kaufman, 1928).

As early as 1884, Weil, noting the motor effects of apomorphine on experimental animals, suggested that the drug might work in PD (Weil, 1884). In 1951, before Carlsson’s discoveries about dopamine, the neurologist Robert Schwab gave apomorphine to parkinsonian patients and observed substantial but short-lived motor responses (Schwab et al., 1951). He had been encouraged by reports that it reduced decerebrate rigidity in animals. There were side-effects of nausea and vomiting, but his paper contains the first clear-cut account of the effects of pharmacological dopamine receptor stimulation in PD. Cotzias, in the years after his levodopa publications, showed that oral apomorphine had strong anti-parkinsonian properties despite side-effects of nausea and apparent renal toxicity (Cotzias et al., 1970). In 1988, in harness with the peripherally-acting dopamine receptor antagonist domperidone to prevent nausea, subcutaneous apomorphine finally become an effective treatment for parkinsonian motor fluctuations (Stibe et al., 1988).

The mid-twentieth century was a heroic era of neuropharmacology. Laboratory catecholamine research was part of the great leap forward in the field of biochemistry. Then there were clinician-researchers epitomized by Schwab and Cotzias, with the imagination and resolve just to try possible treatments and carefully observe the results.

## Ergot alkaloids

**FIGURE 5:**
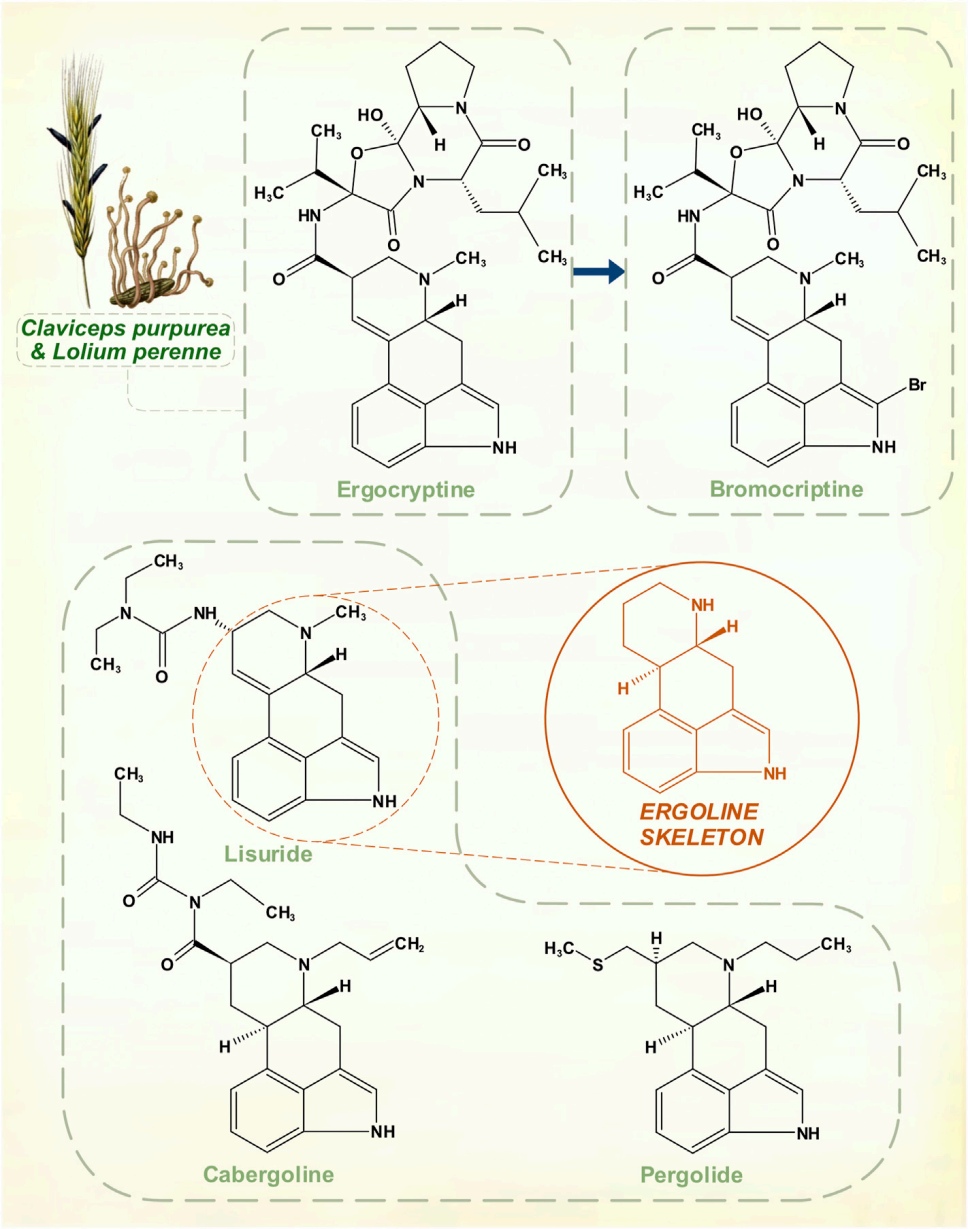
The amino acid L-tryptophan is a precursor for the ergoline skeleton, present in over 70 ergot alkaloids isolated from *Claviceps* fungal species. Naturally occurring ergocryptine and its pharmaceutical modification to create bromocriptine. Other synthetic ergoline dopamine receptor agonists: cabergoline, lisuride and pergolide. Top left: Ergot sclerotia on perennial ryegrass. [Illustrations adapted from Esser (1910) and Köhler (1897), both available in the public domain].

Ergot alkaloids are a group of compounds synthesized from L-tryptophan by members of the Clavicipitaceae family of fungi. The development of semi-synthetic dopamine receptor agonists followed research on the diverse pharmacology of these chemicals, which share a tetracyclic ergoline core (Figure 5). Most interact with adrenergic, dopaminergic and serotonergic receptor systems; relative receptor affinity and balance of full with partial agonism determines their effects (Boissier, 1978). Growth of these fungi in natural forage grasses and domesticated pasture or cereal crops means that herbivorous mammals are often targets of these potent neurotransmitter actions. Human ergotism was usually caused by bread made from fungus-affected rye. The illness could be marked by intense peripheral vasoconstriction, or by hallucinatory delirium (Eadie, 2003).

Suppression of lactation in sows by feed contaminated with fungus had long been recognized in farming. The peptide hormone prolactin was identified as a lactogen in animals during the 1930s (Riddle et al., 1933), and in 1958 it was shown that a mixture of ergocornine, ergocryptine and ergocristine appeared to suppress prolactin in rats (Shelesnyak, 1958). At a time when the existence of prolactin in humans was in still in doubt, Sandoz Pharmaceuticals began to explore potentially marketable compounds in this area. Modifications that could offset oxytocic and vasoconstrictive properties of these alkaloids yet retain the prolactin-lowering action were sought. The result was bromocriptine, a variation on ergocriptine by addition of a single bromine atom to the pyrrole ring of its indole moiety.

Clinical trials on hyperprolactinemic states began before it was understood that dopamine was the inhibitory controller of prolactin secretion (Besser et al., 1972). In 1974, though, bromocriptine proved to be an effective dopamine receptor agonist in the treatment of PD (Calne et al., 1974). Other agents with the ergoline structural core—lisuride, pergolide, cabergoline—were subsequently marketed (Lieberman et al., 1981; Goetz et al., 1983; Lera et al., 1993). None of these drugs was as strong as levodopa, or apomorphine, but their more stable profile of response could be advantageous in patients with motor fluctuations. For this reason, ergoline dopamine receptor agonists, at one point, rivalled the primacy of levodopa. They eventually lost their position because of serotonergic effects that, to a degree, are innate to ergot-derived pharmacological agents. Possibly, this contributed to a tendency to cause neuropsychiatric disturbances in some patients (Parkes et al., 1976). But with prolonged usage, these drugs possess enough agonism at 5-HT_2B_ receptors to stimulate fibroblast activity. This is stronger for pergolide and cabergoline, resulting in dangerous cardiac fibrotic valvulopathies (Antonini and Poewe, 2007).

The *Claviceps* genus, which is associated with human ergotism, affects a range of grasses in a specific manner. The ovaries in grass florets are invaded, with replacement of some seeds in the harvested grain by fungal masses that contain the alkaloids (Haarmann et al., 2009). There is no plant defensive response, yet minimal adverse host effect. The ergot-producing *Epichloë* genus of fungi is endophytic in grasses—hyphae grow in intercellular spaces throughout the above-ground extent of the plant (Clay and Schardl, 2002). These close symbiotic relationships may have been developing for much of the time span of evolution of modern grasses. The predilection for wild and cereal grasses to host ergot-producing fungi suggests that plant-herbivore adaptation is involved.

## Cannabinoids

While William Gowers was dismissive of most inorganic and botanical chemicals used to treat paralysis agitans in the late 19th century, he did put some store in Indian hemp, which he sometimes combined with opium. Several times, he wrote, there was a “very distinct improvement for a considerable time under their use” for tremor (Gowers, 1886).

**FIGURE 6:**
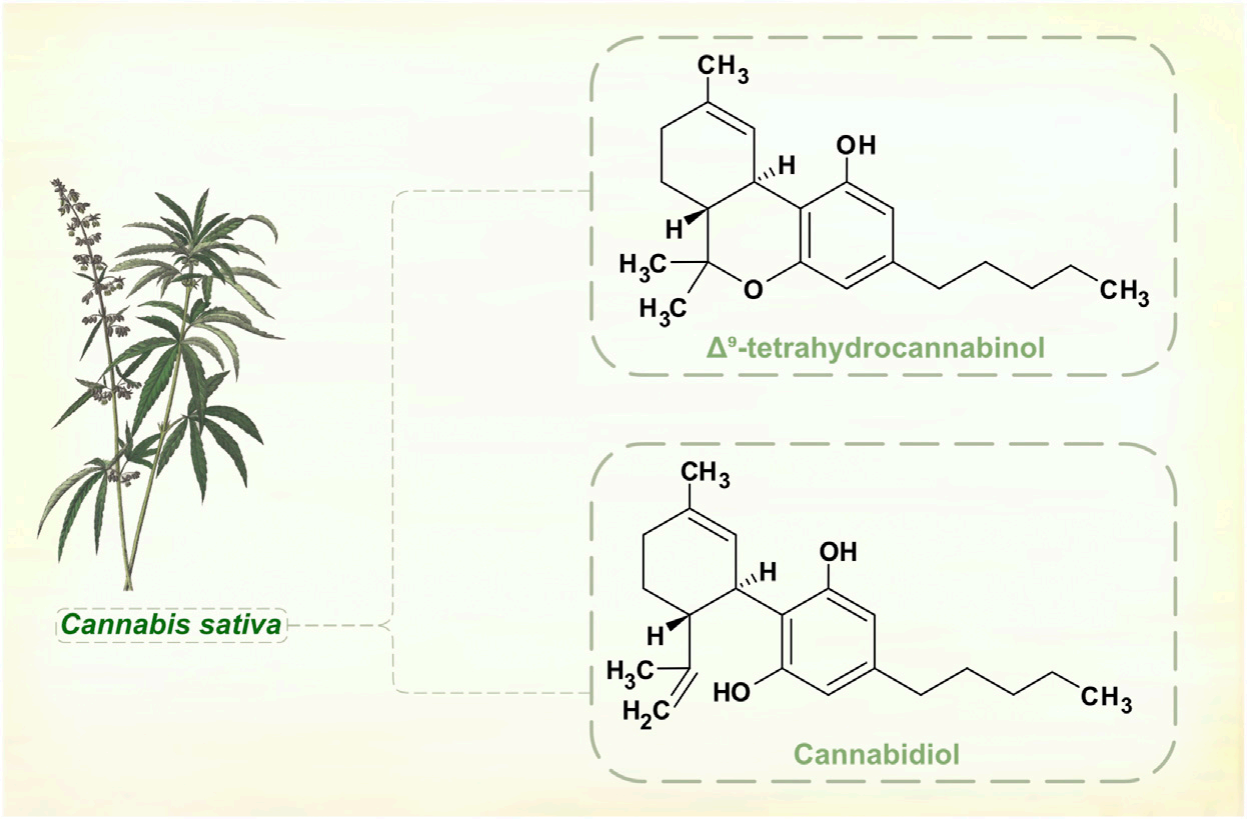
Major phytocannabinoids in *Cannabis sativa*. [Illustration adapted from Yonge (1858), available in the public domain].

Changes in legal status and development of standardized medicinal products has seen cannabis administered for various neurological disorders, including PD. *Cannabis sativa*, *Cannabis indica* and hybrid cultivars grown by the pharmaceutical industry contain a range of cannabinoids. These aromatic compounds have no nitrogen atoms in their molecular structures, and are classed as terpenoids not alkaloids (Figure 6).

Cannabinoids interact with the endocannabinoid neurotransmitter system, and the main psychoactive component of cannabis plants, Δ^9^-tetrahydrocannabinol (THC), is a partial receptor agonist. Secondary actions on dopamine signaling may be important in mediating central nervous system effects of THC, but these are complex and appear to be exposure-dependent (Bloomfield et al., 2016). Acutely administered to laboratory animals, THC enhances dopamine synthesis and release. With repeated dosing, rats show regional differences in brainstem, striatal and cortical dopamine levels, with evidence of both up- and down-regulation. Positron emission tomographic studies suggest that humans who use cannabis regularly have reduced striatal dopamine synthesis (Van de Giessen et al., 2017). There are reports of motor and non-motor benefits in PD. But natural and synthetic cannabinoids have not shown significant effects in controlled clinical trials (Thanabalasingam et al., 2021; Urbi et al., 2022), and there is insufficient evidence for cannabis products as adjunctive dopaminergic therapy.

## Alkaloids—the long game of plants?

Plants have various defensive strategies against herbivorous animals—physical countermeasures such as thick bark, thorns and spikes; and the chemical deterrence afforded by alkaloids and other molecules. The potentially lethal toxicity of tropane alkaloids and strychnine, or the strong emetic effects of *Carapichea ipecacuanha*, easily fit such a model.

A degree of plant-animal co-evolution related to alkaloid production and sensitivity can be inferred, though much of this is recent in relation to the whole timespan of plant ancestry. Angiosperms, the flowering plants, diversified and prospered in the Cretaceous period (145–66 million years ago) (Crane et al., 1995). Gymnosperms (conifers and cycads), which angiosperms displaced as the dominant terrestrial flora, have relatively little expression of alkaloid (Wink, 2020). The phylogeny of insects was greatly influenced by the rise of angiosperms, and many new species developed to interact with plants, both as predators and as agents in plant reproduction. Darwin had puzzled over the eclipse of gymnosperms by angiosperms, and it occurred to him that insect–plant interactions could have led to mutual fitness advantages and accelerated evolution (Darwin, 1862). Alkaloids potentially drive the coevolution of insects and plants through cycles of adaptation then counter adaptation (Ehrlich and Raven, 1964). A plant may develop a toxic alkaloid to deter herbivores, then a selective change to offset the toxicity allows one insect species to prosper. This helps to explain why insect herbivory tends to occur across narrow phylogenetic ranges (insects prefer to feed on one or two plant species, related herbivorous insect species tend to have similar plant preferences).

Living amphibians and reptiles, whose ancestors had survived the dinosaur extinction, are virtually all carnivorous. The herbivory of avians, sole descendants of the dinosaur line, is largely restricted to nectar, soft fruits and nuts. Which leaves mammals as the other main group of animals that feed on plants. In contrast to the narrow preferences of insects, most herbivorous mammals have a broad diet. Modern mammals—plant-eaters, meat-eaters that feed on them, omnivores including primate species—developed from their Cretaceous period ancestors in environments dominated by angiosperms. Mammalian-plant coevolution, though not as extensive as for insects, has determined many phenotypic features—dentition and digestion being only two more obvious examples. Some reflect alkaloid interactions. Detoxifying P450 enzyme systems have adapted to dietary chemical exposure, and herbivorous species like rabbits can inactivate a range of alkaloids. Cats, almost entirely carnivorous, are highly susceptible and can be poisoned by a small quantity of caffeine (Dolder, 2013).

Neuroactive chemicals compose a sizable proportion of plant alkaloids. Targets include acetylcholine, monoamine, amino acid and opioid receptors. Agonists are more common than antagonists, while other alkaloids have effects on neurotransmitter metabolism or storage. Not all conform to a predation discouragement through toxicity model, and some—nicotine, caffeine, morphine, for instance—can reinforce their ingestion (Wink, 2018). Movement and behavior, both in insects and mammals, can potentially be modified. The neurochemistry of motor control in vertebrate brains is a very old evolutionary trait, established well before the appearance of angiosperm plants. Lamprey, primitive jawless fish, diverged from the human lineage 560 million years ago, yet both possess essentially the same basal ganglia circuitry and neurotransmitters (Grillner et al., 2013).

Alkaloid influences on the dopaminergic systems of animals—direct or indirect—are quite common. This could target foraging and feeding activities, with evolutionary effects that highlight plant-animal interdependence. A plant’s fitness can be defined by its ability to maximize the number of viable seeds that it produces. But without spatial distribution of seeds, plants achieve little more than one-for-one replication, giving minimal advantage to new genetic modifications. Seeds can be dispersed by physical forces, or by animals. At the core of their relationship is animals’ requirement for the energy that plants harness, and plants’ need to engage with the animal attribute that they lack—movement. It is likely that plant chemicals have more subtle roles than simply as poisons. Dopamine is strongly associated with energy governance in animals—as a basic enabler of movement, and in budgeting the energy expenditure of motor activity (Beeler et al., 2012). Dopamine has been shown to influence the amount of motor energy that experimental rodents will allocate to gain food reward (Hosking et al., 2015). Alkaloids that interact with dopaminergic systems may be tilting these movement-energy trade-offs in favor of plants.

Alkaloids are a part of a long-standing dialogue between plants and mammals, often conducted in energy-marginal environments. Throughout this time, alkaloids and neurochemical effector systems must have modified one another in various ways. A deep evolutionary relationship helps to explain why the pharmacological treatment of PD is imprinted with the power of these mysterious botanical chemicals.

## References

[B1] Aniszewski T. (2007). Alkaloids - secrets of life. Alkaloid chemistry, biological significance, applications and ecological role. Amsterdam: Elsevier, 61–139.

[B2] Antonini A. Poewe W. (2007). Fibrotic heart-valve reactions to dopamine-agonist treatment in Parkinson's disease. Lancet Neurol. 6, 826–829. 10.1016/S1474-4422(07)70218-1 17706566

[B3] Apaydin H. Ertan S. Ozekmekçi S. (2000). Broad bean (Vicia faba)--a natural source of L-dopa--prolongs "on" periods in patients with Parkinson's disease who have "on-off" fluctuations. Mov. Disord. 15, 164–166. 10.1002/1531-8257(200001)15:1<164::aid-mds1028>3.0.co;2-e 10634260

[B4] Barbeau A. Murphy G. F. Sourkes T. L. (1961). Excretion of dopamine in diseases of basal ganglia. Science 133, 1706–1707. 10.1126/science.133.3465.1706-a 13686753

[B5] Barbeau A. Sourkes T. L. Murphy G. F. (1962). “Les catécholamines dans la maladie de Parkinson,” in Monoamines et Système Nerveux Central. Editor de Ajuriaguerra J. (Geneva: Georg & Cie), 247–262.

[B6] Barriga Villalba A. M. (1925). Yajéine. A new alkaloid. J. Soc. Chem. Ind. 44, 205–207.

[B7] Beeler J. A. Frazier C. R. M. Zhuang X. (2012). Putting desire on a budget: Dopamine and energy expenditure, reconciling reward and resources. Front. Integr. Neurosci. 6, 49. 10.3389/fnint.2012.00049 22833718 PMC3400936

[B8] Beringer K. (1928). Über ein neues, auf das extrapyramidal-motorische System wirkendes Alkaloid (Banisterin). Nervenarzt 5, 20–30.

[B9] Bertol E. Fineschi V. Karch S. B. Mari F. Riezzo I. (2004). Nymphaea cults in ancient Egypt and the new world: A lesson in empirical pharmacology. J. R. Soc. Med. 97, 84–85. 10.1258/jrsm.97.2.84 14749409 PMC1079300

[B10] Besser G. M. Parke L. Edwards C. R. Forsyth I. A. McNeilly A. S. (1972). Galactorrhoea: Successful treatment with reduction of plasma prolactin levels by brom-ergocryptine. Br. Med. J. 3, 669–672. 10.1136/bmj.3.5828.669 4675488 PMC1786102

[B11] Bharate S. S. Mignani S. Vishwakarma R. A. (2018). Why are the majority of active compounds in the CNS domain natural products? A critical analysis. J. Med. Chem. 61, 10345–10374. 10.1021/acs.jmedchem.7b01922 29989814

[B12] Birkmayer W. Hornykiewicz O. (1961). Der L-3,4-Dioxyphenlalanin (L-Dopa) – effekt bei der Parkinson-Akinese. Wien. Klin. Wochenschr. 73, 787–788. 13869404

[B13] Bloomfield M. A. P. Ashok A. H. Volkow N. D. Howes O. D. (2016). The effects of Δ9-tetrahydrocannabinol on the dopamine system. Nature 539, 369–377. 10.1038/nature20153 27853201 PMC5123717

[B14] Boissier J. R. (1978). General pharmacology of ergot alkaloids. Pharmacology 16 (1), 12–26. 10.1159/000136805 205888

[B15] Brabec de Mori B. (2011). “Tracing hallucinations: Contributing to a critical ethnohistory of ayahuasca usage in the Peruvian Amazon,” in The internationalization of ayahuasca. Editors Labate, B. C. Jungaberle H. (Zürich: Lit Verlag), 23–47.

[B16] Brodie B. B. Pletscher A. Shore P. A. (1955). Evidence that serotonin has a role in brain function. Science 122, 968. 10.1126/science.122.3177.968 13274056

[B17] Bulliard P. (1780). Herbier de la France; ou, Collection complette des plantes indigènes de ce royaume; avec leurs détails anatomique, leurs propriétés et leurs usages en médecine. Paris: Chez l'auteur.

[B18] Calne D. B. Teychenne P. F. Claveria L. E. Eastman R. Greenacre J. K. Petrie A. (1974). Bromocriptine in parkinsonism. Br. Med. J. 4, 442–444. 10.1136/bmj.4.5942.442 4425916 PMC1612580

[B19] Carlsson A. Lindqvist M. Magnusson T. (1957). 3,4-dihydroxyphenylalanine and 5-hydroxytryptophan as reserpine antagonists. Nature 180, 1200. 10.1038/1801200a0 13483658

[B20] Carlsson A. (1959). The occurrence, distribution and physiological role of catecholamines in the nervous system. Pharmacol. Rev. 11, 490–493. 13667431

[B21] Charcot J-M. (1877). “On Parkinson's disease,” in Lectures on the diseases of the nervous system. Delivered at La salpêtrière, Trans G. Sigerson (London: The New Sydenham Society), 155–156.

[B22] Clay K. Schardl C. (2002). Evolutionary origins and ecological consequences of endophyte symbiosis with grasses. Am. Nat. 160, S99–S127. 10.1086/342161 18707456

[B23] Cotzias G. C. Van Woert M. H. Schiffer L. M. (1967). Aromatic amino acids and modification of Parkinsonism. N. Engl. J. Med. 276, 374–379. 10.1056/NEJM196702162760703 5334614

[B24] Cotzias G. C. Papavasiliou P. S. Gellene R. (1969). Modification of parkinsonism--chronic treatment with L-dopa. N. Engl. J. Med. 280, 337–345. 10.1056/NEJM196902132800701 4178641

[B25] Cotzias G. C. Papavasiliou P. S. Fehling B. Kaufman I. (1970). Similarities between neurologic effects of L-Dopa and apomorphine. N. Engl. J. Med. 282, 31–33. 10.1056/NEJM197001012820107 4901383

[B26] Crane P. R. Friis E. M. Pedersen K. R. (1995). The origin and early diversification of angiosperms. Nature 374, 27–33. 10.1038/374027a0

[B27] Curtis W. Hooker J. D. Hooker W. J. S. Prain D. Stapf O. Bentham-Moxon T. (1822). Curtis's botanical magazine. London: Academic Press.

[B28] Darwin C. (1862). On the various contrivances by which British and foreign orchids are fertilised by insects: And on the good effects of intercrossing. London: John Murray. PMC5180317 30163543

[B29] de Boodt A. B. (1596-1610). Holwortel (Corydalis cava). Delft.

[B30] De N. (1944/1945). Neurological and mental symptoms produced by the therapeutic dose of Rauwolfia serpentina and mepacrine hydrochloride. Trans. Med. Coll. Reun. (Calcutta) 7, 27–29.

[B31] Degkwitz R. Frowein R. Kulenkampff C. Mohs U. (1960). Über die Wirkungen des L-Dopa beim Menschen und deren Beeinflussung durch Reserpin, Chlorpromazine, Iproniazid und Vitamin B_6_ . Klin. Wochenschr. 38, 120–123. 10.1007/bf02189076 13815400

[B32] Dent J. Y. (1934). Apomorphine in the treatment of anxiety states, with special reference to alcoholism. B. J. Inebr. 32, 65–88. 10.1111/j.1360-0443.1934.tb05016.x

[B33] Djamshidian A. Poewe W. (2016). Apomorphine and levodopa in Parkinson's disease: Two revolutionary drugs from the 1950's. Park. Relat. Disord. 33, S9–S12. 10.1016/j.parkreldis.2016.12.004 28012951

[B34] Dolder L. K. (2013). “Methylxanthines: Caffeine, theobromine, theophylline,” in Small animal toxicology. Editors Peterson, D. Talcott P. (St. Louis, Missouri: Elsevier Saunders), 647–652. 10.1016/b978-1-4557-0717-1.00060-0

[B35] Dole V. P. (1996). George Constantin Cotzias. Biographical Memoirs, 68. Washington D.C.: National Academy of Sciences, 63–70. 11616358

[B36] Doshay L. Constable K. Zier A. (1954). Five year follow-up of treatment with trihexyphenidyl (Artane); outcome in four hundred eleven cases of paralysis agitans. J. Am. Med. Assoc. 154, 1334–1336. 10.1001/jama.1954.02940500014005 13151847

[B37] Eadie M. J. (2003). Convulsive ergotism: Epidemics of the serotonin syndrome? Lancet Neurol. 2, 429–434. 10.1016/s1474-4422(03)00439-3 12849122

[B38] Ehringer H. Hornykiewicz O. (1960). Verteilung von Noradrenalin und Dopamin (3-Hydroxytyramin) im Gehirn des Menschen und ihr Verhalten bei Erkrankungen des extrapyramidalen Systems. Klin. Wochenschr. 38, 1236–1239. 10.1007/bf01485901 13726012

[B39] Ehrlich P. R. Raven P. H. (1964). Butterflies and plants: A study in coevolution. Evolution 18, 586–608. 10.1111/j.1558-5646.1964.tb01674.x

[B40] Embolden W. A. (1982). The mushroom and the waterlily: Literary and pictorial evidence for Nymphaea as a ritual psychoten in mesoamerica. J. Ethnopharmacol. 5, 139–148. 10.1016/0378-8741(82)90039-33 7035751

[B41] Erb W. H. (1887). Über Hyoscin. Therap. Monatshefte, 1, 252–254.

[B42] Esser P. (1910). Die giftpflanzen deutschlands. Braunschweig: Friedr, Vieweg & Sohn.

[B43] Fahn S. (1974). On-off' phenomenon with levodopa therapy in parkinsonism. Neurology 24, 431–441. 10.1212/wnl.24.5.431 4857104

[B44] Fehling C. (1966). Treatment of Parkinson's syndrome with L-Dopa: A double blind study. Acta. Neurol. Scand. 42, 367–372. 10.1111/j.1600-0404.1966.tb01188.x 5327616

[B45] Fleischhacker H. (1926). Ueber den Einfluß des Bulbocapnium hydrochloricum auf verschiedene Hyperkinesen. Dtsch. Med. Wochenschr. 52, 362. 10.1055/s-0029-1200755

[B46] Flury F. (1911). Beiträge zur Pharmakologie der Steppenraute (Perganum hamala). Arch. Exp. Pathol. Pharmakol. 64, 105–125. 10.1007/BF01840797

[B47] Foley P. B. (2003). Beans, roots and leaves: A history of the chemical therapy of parkinsonism. Marburg: Tectum Verlag. 15641199

[B48] Fothergill-Misbah N. Maroo H. Cham M. Pezzoli G. Walker R. Cilia R. (2020). Could Mucuna pruriens be the answer to Parkinson's disease management in sub-Saharan Africa and other low-income countries worldwide? Park. Relat. Disord. 73, 3–7. 10.1016/j.parkreldis.2020.03.002 32179240

[B49] Frank H. Schlesinger O. (1930). Klinische Erfahrungen bei der Behandlung der Postencephalitischen Erscheinungen mit. Harmin. Klin. Wochenschr. 9, 1864–1866. 10.1007/bf01737859

[B50] Fritzsche J. (1847). Bestandtheile der Samen von Perganum hamala. Justus Liebigs Ann. Chem. 64, 360–364.

[B51] Galili G. Amir R. Fernie A. R. (2016). The regulation of essential amino acid synthesis and accumulation in plants. Annu. Rev. Plant. Biol. 67, 153–178. 10.1146/annurev-arplant-043015-112213 26735064

[B52] Geiger H. (1833). Darstellung des Atropins. Ann. Phar. 5, 43–81. 10.1002/jlac.18330050108

[B53] Goebel F. (1841). Ueber das Harmalin. Justus Liebigs Ann. Chem. 38, 363–366. 10.1002/jlac.18410380318

[B54] Goetz C. G. Tanner C. M. Glantz R. Klawans H. L. (1983). Pergolide in Parkinson's disease. Arch. Neurol. 40, 785–787. 10.1001/archneur.1983.04050120035003 6639407

[B55] Goodman L. Gilman A. (1955). The pharmacological basis of therapeutics: A textbook for physicians and medical students. 2nd edn. New York: Macmillan, 552–553.

[B56] Gowers W. R. (1886). A Manual of Diseases of the Nervous System, 2. London: J & A Churchill, 607.

[B57] Grillner S. Robertson B. Stephenson-Jones M. (2013). The evolutionary origin of the vertebrate basal ganglia and its role in action selection. J. Physiol. 591, 5425–5431. 10.1113/jphysiol.2012.246660 23318875 PMC3853485

[B58] Guggenheim M. (1913). Dioxyphenylalanin: Eine neue aminosäure aus Vicia faba. Z. Physiol. Chem. 88, 276–284. 10.1515/bchm2.1913.88.4.276

[B59] Guggenheim M. (1961). Historische Betrachtungen über Dioxyphenylalanin, Catecholamine und andere biogene Amine. Bull. Schweiz. Akad. Med. Wiss. 17, 309–316. 13902754

[B60] Haarmann T. Rolke Y. Giesbert S. Tudzynski P. (2009). Ergot: From witchcraft to biotechnology. Mol. Plant Pathol. 10, 563–577. 10.1111/j.1364-3703.2009.00548.x 19523108 PMC6640538

[B61] Halpern L. (1931). Grundsätzliches zu einer Differentialtherapie des Parkinsonsyndroms. Klin. Wochenschr. 10, 983–985. 10.1007/bf01748241

[B62] Harnack E. (1874). Ueber die Wirkungen des Apomorphins am Säugethier und am Frosch. Arch. Exp. Pathol. Pharmakol. 2, 254–306. 10.1007/bf01976871

[B63] Harris T. H. (1957). Depression induced by Rauwolfia compounds. Am. J. Psychiatry 113, 950. 10.1176/ajp.113.10.950 13402993

[B64] Hosking J. G. Floresco S. B. Winstanley C. A. (2015). Dopamine antagonism decreases willingness to expend physical, but not cognitive, effort: A comparison of two rodent cost/benefit decision-making tasks. Neuropsychopharmacol 40, 1005–1015. 10.1038/npp.2014.285 PMC4330516 25328051

[B65] Jain S. Murthy P. (2009). The other bose: An account of missed opportunities in the history of neurobiology in India. Curr. Sci. 97, 266–269.

[B66] Katzenschlager R. Evans A. Manson A. Patsalos P. N. Ratnaraj N. Watt H. (2004). Mucuna pruriens in Parkinson's disease: A double blind clinical and pharmacological study. J. Neurol. Neurosurg. Psychiatry 75, 1672–1677. 10.1136/jnnp.2003.028761 15548480 PMC1738871

[B67] Kempster P. A. Bogetic Z. Secombe J. W. Martin H. D. Balazs N. D. H. Wahlqvist M. L. (1993). Motor effects of broad beans (Vicia faba) in Parkinson's disease: Single dose studies. Asia Pac. J. Clin. Nutr. 2, 85–89. 24352104

[B68] Kline N. S. Stanley A. M. (1955). Use of reserpine in a neuropsychiatric hospital. Ann. N. Y. Acad. Sci. 61, 85–91. 10.1111/j.1749-6632.1955.tb42454.x 14377275

[B69] Köhler H. (1897). “Köhler’s Medizinal-Planzen,” in Naturgetreuen Abbildungen mit kurz erläuterndem Texte: Atlas zur Pharmacopoea germanica, austriaca, belgica, danica, helvetica, hungarica, rossica, suecica, Neerlandica, British pharmacopoeia, zum Codex medicamentarius, sowie zur Pharmacopoeia of the United States of America Editor Pabst G. (Gera: Verlag von Fr. Eugen Köhler). 10.5962/bhl.title.623

[B70] Ladenburg A. (1880a). Über das Hyosin. Ber. Dtsch. Chem. Ges. 13, 1549–1554. 10.1002/cber.18800130273

[B71] Ladenburg A. (1880b). Beziehungen zwischen Hyoscyamin und Atropin und Verwandlung des einen Alkaloids in das andere. Ber. Dtsch. Chem. Ges. 13, 607–609. 10.1002/cber.188001301166

[B72] Leiner J. Kaufman R. (1928). Bulbocapnine in disease manifesting dyskinesia. Clinical and therapeutic observations in nineteen cases divided into groups. AMA Arch. Neurol. Psychiatry 20, 1269–1283. 10.1001/archneurpsyc.1928.02210180120006

[B73] Lera G. Vaamonde J. Rodriguez M. Obeso J. A. (1993). Cabergoline in Parkinson's disease: Long-term follow-up. Neurology 43, 2587–2590. 10.1212/wnl.43.12.2587 7902970

[B74] Lewin L. (1928). Sur une substance enivrante, la Banisterine, extraite de Banisteria caapi. C. R. Acad. Sci. Paris. 186, 469–471.

[B75] Lieberman A. Goldstein M. Neophytides A. Kupersmith M. Leibowitz M. Zasorin N. (1981). Lisuride in Parkinson disease: Efficacy of lisuride compared to levodopa. Neurology 31, 961–965. 10.1212/wnl.31.8.961 7022259

[B76] Löffler W. (1971). Markus guggenheim, 1885-1970. Bull. Schweiz. Akad. Med. Wiss. 27, 167–169. 4946625

[B77] López-Muñoz F. Alamo C. Cuenca E. Shen W. W. Clervoy P. Rubio G. (2005). History of the discovery and clinical introduction of chlorpromazine. Ann. Clin. Psychiatry 17, 113–135. 10.1080/10401230591002002 16433053

[B78] Marsden C. D. Parkes J. D. (1977). Success and problems of long-term levodopa therapy in Parkinson's disease. Lancet 309 (8007), 345–349. 10.1016/S0140-6736(77)91146-1 64868

[B79] McGeer P. L. Boulding J. E. Gibson W. C. Foulkes R. G. (1961). Drug-induced extrapyramidal reactions: Treatment with diphenhydramine hydrochloride and dihydroxyphenylalanine. J. Am. Med. Assoc. 177, 665–670. 10.1001/jama.1961.03040360001001 13773933

[B80] Mcgeer P. L. Zeldowicz L. R. (1964). Administration of dihydroxyphenylalanine to parkinsonian patients. Can. Med. Assoc. J. 90, 463–466. 14120951 PMC1922247

[B81] McKenna D. J. Callaway J. C. Grob C. S. (1998). The scientific investigation of ayahuasca: A review of past and current research. Heffter Rev. Psyched. Res. 1, 65–76.

[B82] Melvin E. Daxenbichler C. H. Etten V. Earle F. R. Tallent W. H. (1972). L-Dopa recovery from Mucuna seed. J. Agric. Food Chem. 20, 1046–1048. 10.1021/jf60183a002 5057437

[B83] Müller J. M. Schlittlet E. Bein H. J. (1952). Reserpin, der sedative Wirkstoff aus Rauwolfia serpentina Benth. Experientia 8, 338. 10.1007/BF02174406 12998611

[B84] Near J. A. Mahler H. R. (1983). Reserpine labels the catecholamine transporter in synaptic vesicles from bovine caudate nucleus. FEBS Lett. 158, 31–35. 10.1016/0014-5793(83)80670-x 6862032

[B85] Neumeyer J. L. (1985). “Synthesis and structure-activity relationships of aporphines as dopamine receptor agonists and antagonists,” in The chemistry and biology of isoquinoline alkaloids. Editors Phillipson J. D. Roberts M. F. Zenk M. H. (Berlin: Springer-Verlag), 146–170. 10.1007/978-3-642-70128-3_10

[B86] Neuwahl F. J. Fenwick C. C. (1937). Bulgarian treatment of post-encephalitic parkinsonism. Lancet 230 (5950), 619–621. 10.1016/s0140-6736(00)88585-2

[B87] Ordenstein L. (1867). Sur la paralysie agitante et la sclérose en plaques généralisée. Paris: E. Martinet, 31.

[B88] Parkes J. D. Debono A. G. Marsden C. D. (1976). Bromocriptine in parkinsonism: Long-lerm treatment, dose response, and comparison with levodopa. J. Neurol. Neurosurg. Psychiatry 39, 1101–1108. 10.1136/jnnp.39.11.1101 1036999 PMC1083310

[B89] Pelletier S. W. (1983). “The nature and definition of an alkaloid,”. Alkaloids: Chemical and biological perspectives. Editor Pelletier S. W. (New York: Wiley), 1, 1–31.

[B90] Poklis J. L. Mulder H. A. Halquist M. S. Wolf C. E. Poklis A. Peace M. R. (2017). The blue lotus flower (Nymphea caerulea) resin used in a new type of electronic cigarette, the re-buildable dripping atomizer. J. Psychoact. Drugs 49, 175–181. 10.1080/02791072.2017.1290304 PMC5638439 28266899

[B91] Redouté P. (1833). in Choix des plus belles fleurs et des plus beaux fruits. Editor Panckoucke E. (Paris: Chez l'auteur).

[B92] Richman A. Tyhurst J. S. (1955). An extrapyramidal syndrome with reserpine. Can. Med. Assoc. J. 72, 457–458. 14352118 PMC1825448

[B93] Riddle O. Bates R. Dykshorn S. (1933). The preparation, identification and assay of prolactin - a hormone of the anterior pituitary. Am. J. Physiol. 105, 191–216. 10.1152/ajplegacy.1933.105.1.191

[B94] Römer C. (1931). Die Atropinbehandlung der encephalitischen Folgezustände. Zfdg. Neuro. U. Psych. 132, 724–736. 10.1007/BF02863875

[B95] Römer C. (1932). Zur Atropinbehandlung des Parkinsonismus. Med. Welt. 6, 1127–1129.

[B96] Rustige E. (1929). Versuche mit Harmin bei Metenzephalitikern. Dtsche. Med. Wochenschr. 15, 613–614. 10.1055/s-0028-1126437

[B97] Scherman D. Henry J. P. (1984). Reserpine binding to bovine chromaffin granule membranes. Characterization and comparison with dihydrotetrabenazine binding. Mol. Pharmacol. 25, 113–122. 6708929

[B98] Schmidt E. Henschkem H. (1888). Über die Alkaloide der Wurzel von Scopolia japonica. Arch. Pharm. 226, 185–203. 10.1002/ardp.18882260502

[B99] Schwab R. S. Amador R. Levine J. Y. (1951). Apomorphine in Parkinson's disease. Trans. Am. Neurol. Assoc. 76, 251–253. 14913646

[B100] Selmar D. Kleinwächter M. (2013). Stress enhances the synthesis of secondary plant products: The impact of stress-related over-reduction on the accumulation of natural products. Plant. Cell. Physiol. 54, 817–826. 10.1093/pcp/pct054 23612932

[B101] Shelesnyak M. C. (1958). Maintenance of gestation of ergotoxine-treated pregnant rats by exogenous prolactin. Acta. Endocrinol. 27, 99–109. 10.1530/acta.0.0270099 13497524

[B102] Shepherd M. Watt D. C. (1956). A controlled clinical study of chlorpromazine and reserpine in chronic schizophrenia. J. Neurol. Neurosurg. Psychiatry 19, 232–235. 10.1136/jnnp.19.3.232 13357965 PMC497211

[B103] Shore P. A. Silver S. L. Brodie B. B. (1955). Interaction of reserpine, serotonin, and lysergic acid diethylamide in brain. Science 122, 284–285. 10.1126/science.122.3163.284-a 13246633

[B104] Stibe C. M. H. Lees A. J. Kempster P. A. Stern G. M. (1988). Subcutaneous apomorphine in parkinsonian on-off oscillations. Lancet 331 (8582), 403–406. 10.1016/s0140-6736(88)91193-2 2893200

[B105] Strong A. B. (1855a). The American Flora, 1. New York: Strong and Burdick.

[B106] Strong A. B. (1855b). The American Flora, 2. New York: Strong and Burdick.

[B107] Strong A. B. (1855c). The American Flora, 3. New York: Strong and Burdick.

[B108] Taba P. Lees A. J. Stern G. (2013). Erich Harnack (1852–1915) and a short history of apomorphine. Eur. Neurol. 69, 321–324. 10.1159/000346762 23549143

[B109] Thanabalasingam S. J. Ranjith B. Jackson R. Wijeratne D. T. (2021). Cannabis and its derivatives for the use of motor symptoms in Parkinson’s disease: A systematic review and meta-analysis. Ther. Adv. Neurol. Disord. 14, 1–22. 10.1177/17562864211018561 PMC8161868 34104218

[B110] Thomé O. W. (1885). Flora von deutschland, österreich und der schweiz. Eugen Köhler: Gera-Untermhaus: Verlag von Fr.

[B111] Tohge T. Watanabe M. Hoefgen R. Fernie A. (2013). Shikimate and phenylalanine biosynthesis in the green lineage. Front. Plant. Sci. 4, 62. 10.3389/fpls.2013.00062 23543266 PMC3608921

[B112] Udenfriend S. Witkop B. Redfield B. G. Weissbach H. (1958). Studies with reversible inhibitors of monoamine oxidase: Harmaline and related compounds. Biochem. Pharm. 1, 160–165. 10.1016/0006-2952(58)90025-x

[B113] Urbi B. Corbett J. Hughes I. Owusu M. A. Thorning S. Broadley S. (2022). Effects of cannabis in Parkinson’s disease: A systematic review and meta-analysis. J. Park. Dis. 12, 495–508. 10.3233/JPD-212923 34958046

[B114] Van de Giessen E. Weinstein J. J. Cassidy C. M. Haney M. Dong Z. Ghazzaoui R. (2017). Deficits in striatal dopamine release in cannabis dependence. Mol. Psychiatry. 22, 68–75. 10.1038/mp.2016.21 27001613 PMC5033654

[B115] Van Rheede tot Drakenstein H. (1686). Indian snakeroot or java devilpepper (rauvolfia serpentina (L.) kurz): Flowering and fruiting branches, root, inflorescence and sectioned fruit with seeds. Amsterdam.

[B116] Waller G. R. Nowacki E. K. (1978). Alkaloid biology and metabolism in plants. London & New York: Plenum Press, 121–141. 10.1007/978-1-4684-0772-3_4

[B117] Weil E. (1884). De l’apomorphine dans certain troubles nerveux. Lyon Méd 48, 411–419.

[B118] Wilson S. A. K. (1940). Neurology, 2. Baltimore: Williams & Wilkins, 804–805. 10.1136/bmj.2.4170.805

[B119] Wink M. (2003). Evolution of secondary metabolites from an ecological and molecular phylogenetic perspective. Phytochemistry 64, 3–19. 10.1016/s0031-9422(03)00300-5 12946402

[B120] Wink M. (2018). Plant secondary metabolites modulate insect behavior—Steps toward addiction? Front. Physiol. 9, 364. 10.3389/fphys.2018.00364 29695974 PMC5904355

[B121] Wink M. (2020). “Evolution of the angiosperms and co-evolution of secondary metabolites, especially of alkaloids,” in Co-evolution of secondary metabolites. Editors Mérillon, J-M. Ramawat K. G. (Cham (Switzerland): Springer Nature Switzerland AG), 151–174. 10.1007/978-3-319-96397-6_22

[B122] Wolfes O. Ivers O. (1929). Über Harmin. Ein mit dem Bannisterin (Yagein) identisches Alkaloid. I. Chemischer Teil. Merks Jahresber. über Neuer. den Gebeiten Pharmakother. 42, 5.

[B123] Yalcin D. Bayraktar O. (2010). Inhibition of catechol-O-methyltransferase (COMT) by some plant-derived alkaloids and phenolics. J. Mol. Catal. B. Enzym. 64, 162–166. 10.1016/j.molcatb.2009.04.014

[B124] Yonge C. M. (1858). The instructive picture book, or, Lessons from the vegetable world. Editor Stark R. M. (Edinburgh: Edmonston & Douglas).

